# Potentiodynamic and Galvanodynamic Regimes of Mass Transfer in Flow-Through Electrodialysis Membrane Systems: Numerical Simulation of Electroconvection and Current-Voltage Curve

**DOI:** 10.3390/membranes10030049

**Published:** 2020-03-20

**Authors:** Aminat Uzdenova, Makhamet Urtenov

**Affiliations:** 1 Department of Computer Science and Computational Mathematics, Federal State Budgetary Educational Institution of Higher Education “Umar Aliev Karachai-Cherkess State University”, 369202 Karachaevsk, Russia; 2 Department of Applied Mathematics, Federal State Budgetary Educational Institution of Higher Education “Kuban State University”, 350040 Krasnodar, Russia; urtenovmax@mail.ru

**Keywords:** ion-exchange membrane, electrodialysis, current-voltage curve, electroconvection, potentiodynamic regime, galvanodynamic regime, numerical simulation

## Abstract

Electromembrane devices are usually operated in two electrical regimes: potentiodynamic (PD), when a potential drop in the system is set, and galvanodynamic (GD), when the current density is set. This article theoretically investigates the current-voltage curves (CVCs) of flow-through electrodialysis membrane systems calculated in the PD and GD regimes and compares the parameters of the electroconvective vortex layer for these regimes. The study is based on numerical modelling using a basic model of overlimiting transfer enhanced by electroconvection with a modification of the boundary conditions. The Dankwerts’ boundary condition is used for the ion concentration at the inlet boundary of the membrane channel. The Dankwerts’ condition allows one to increase the accuracy of the numerical implementation of the boundary condition at the channel inlet. On the CVCs calculated for PD and DG regimes, four main current modes can be distinguished: underlimiting, limiting, overlimiting, and chaotic overlimiting. The effect of the electric field regime is manifested in overlimiting current modes, when a significant electroconvection vortex layer develops in the channel.

## 1. Introduction

**Figure 1:**
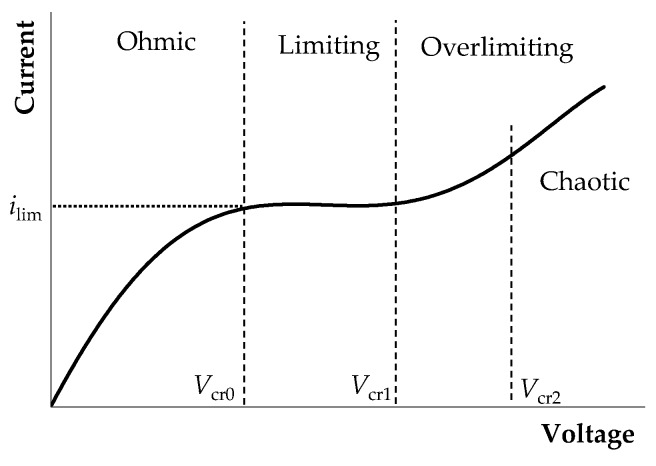
Sketch of a typical current-voltage curve (CVC) of an ion-exchange membrane. The dashed lines *V*_cr0_, *V*_cr1_, *V*_cr2_ indicate changes in the CVC regions: underlimiting current, plateau of the limiting current (*i*_lim_), overlimiting, overlimiting with chaotic oscillations.

**Figure 2:**
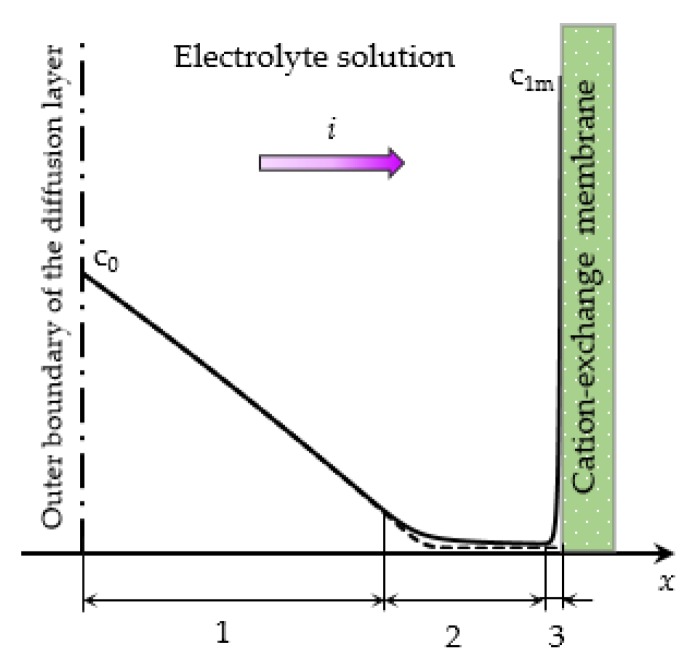
Schematic concentration profiles of cations (*c*_1_, the solid line) and anions (*c*_2_, the dashed line) in the diffusion layer adjacent to the surface of a cation-exchange membrane (CEM) [25]. The current density *i* is flowing across the system; the electrolyte concentration in the bulk solution, *c*_0_; the cation concentration at the solution/CEM boundary, *c*_1m_; different diffusion layer regions are shown: the electroneutral region (**1**), the extended SCR (**2**) and the quasi-equilibrium electric double layer (**3**), respectively.

**Figure 3:**
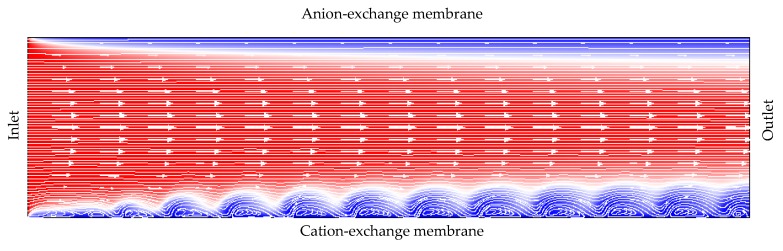
Scheme of the flow of an electrolyte solution in the channel between the anion-exchange and cation-exchange membranes, with taking into account the forced flow (shown by arrows) and the development of an electroconvective vortex layer (at the CEM surface). Ion depletion zones are shown in blue. Based on [19].

Flow-through electrodialysis (ED) membrane cells are widely used in water purification and the processing of agricultural products (milk, wine, etc.) [1,2,3,4]. Electromembrane systems are described by a nonlinear current-voltage curve (CVC), owing largely to the phenomena of concentration polarization, current-induced convection, and water dissociation [5,6]. For dilute electrolyte solutions considered in this article, the main mechanism of overlimiting transfer is electroconvection, as shown by experimental [7,8,9,10,11,12,13] and theoretical studies [14,15,16,17,18,19]. It is customary to distinguish three modes on the CVC of membrane system (Figure 1):

(1) The underlimiting current (ohmic behavior) is the initial linear region of the CVC, which is characterized by a rather high concentration of ions in the region near the membrane. When an electric current flows through the ion-exchange membrane, the ion concentration decreases on one side of the membrane and increases on the other due to the selective transfer of counterions in the membrane (ion concentration polarization). With the increase in the potential drop, almost complete depletion of ions in the region at the membrane surface in the channel of desalination and the transition of the system to the limiting state are observed [20,21].
(2) The limiting current is a section of the CVC with a small slope (plateau), which describes the saturation of the current corresponding to the almost complete depletion of ions at the membrane surface [22,23].
(3) The overlimiting current is the region of secondary current growth: with a further increase in the applied potential drop, the current takes on values greater than the limiting. The increase in the electric current essentially indicates an increase in the conductivity of the depleted region. For dilute electrolyte solutions, electroconvection is the main process that partially destroys the depleted region [7,8,9,10,11,12,13,14,15,16,17,18,19]. Electroconvection is the entrainment of liquid molecules by ions that form a space charge at the ion-selective surface under the influence of the electric force [24]. The intensity of electroconvection increases significantly with the passage of the overlimiting current when an extended macroscopic space charge region (SCR) is formed at the interface due to the polarization of the electric double layer (EDL) (Figure 2 and Figure 3).

The existence of the extended SCR at the electrolyte solution/membrane interface, which is much larger than the region of the equilibrium EDL when a sufficiently high voltage is applied, was first shown by I. Rubinstein and L. Shtilman on the basis of a numerical solution of the Nernst-Planck and Poisson equations for the potential of the electric field [25].

Later, I. Rubinstein and B. Zaltzman [14] developed a model for describing mass transfer in a diffusion layer at a homogeneous ion-exchange membrane. They found a numerical solution for the Nernst-Planck-Poisson and Navier-Stokes equations under the assumption of local electroneutrality in the solution outside the SCR and using a special condition of electroosmotic slip at the interface of the electroneutral region with the SCR. It was shown that the heterogeneity of the surface is not a necessary condition for the emergence of electroconvection. A characteristic feature of this system is its hydrodynamic instability at sufficiently high potential drop. Several threshold potential drops were established, which separate different phases in the development of electrokinetic instability.

Approaches to electroconvection modelling using the slip condition at the boundary with the SCR were applied by V. Dydek et al. [26], R. Abu-Rjal et al. [27]. Models based on the Nernst-Planck-Poisson and Navier-Stokes equations that directly take into account the formation of the extended SCR were considered in the works of E.A. Demekhin et al. [15,28,29], S.V. Pham et al. [16,30], and K. Druzgalski, E. Karatay et al. [18,31], P. Magnico [32,33]. Numerical studies of electroconvection flows generated at an electrically heterogeneous membrane surface were carried out by S. Davidson et al. [34], M. Andersen et al. [35], V.A. Kirii et al. [36].

The difference in the CVCs of the ion-exchange membrane without a forced flow during the transition between the limiting and overlimiting current regimes at the increasing and decreasing potential drop was theoretically described in [11,16,28,32]. S.V. Pham et al. examined a wavy membrane and explained hysteretic behavior by the fact that in the decreasing regime the existing depletion zone creates a lateral gradient, which creates a high lateral electric field. Thus, an additional lateral volumetric force is created to maintain the vortex flow. E.A. Demekhin et al. [28] investigated the hysteresis behavior of ideal smooth ion-exchange membranes and showed the dependence of the hysteresis amplitude on the coupling coefficient between the hydrodynamics and the electrostatics. Hysteretic amplitude calculations observed by Demekhin et al. has been confirmed by P. Magnico [32].

Two electroconvection kinds can be distinguished in overlimiting current modes in membrane systems. In the case of a curved or electrically heterogeneous surface, the tangential electric field causes a stable electroosmotic transfer, described in the works of S.S. Dukhin and N.A. Mishchuk [37,38,39]. In the case of homogeneous membranes in the absence of forced fluid flow, such a kind is not realized: electroconvection appears as a result of hydrodynamic instability, as shown by I. Rubinstein and B. Zaltzman [14]. These two kinds are sometimes termed as electrokinetic modes, respectively, of Dukhin and Rubinstein [40].

Electroconvection in ED channels with forced fluid flow was investigated by M.Kh. Urtenov et al. [19,41] and R. Kwak et al. [17,42], R. Abu-Rjal et al. [27], P. Magnico [33]. In such channels, the concentration is distributed unevenly along the length of the channel: as the solution moves between the membranes, the electrolyte concentration decreases and the thickness of the diffusion layer increases. In this case, a tangential bulk electric force is formed, that acts on the SCR at the depleted surface of the membrane, even if the membrane is homogeneous. This force causes stationary electroconvection even at underlimiting current densities. According to the terminology of S.S. Dukhin and N.A. Mishchuk [37,38,39], this type of electroconvection can be considered as an electroosmosis of the first kind. The bulk force is localized at a relatively small distance from the membrane, where viscous forces play an important role due to the adhesion condition. The contribution of electroconvection to the increase of current becomes significant only at the potential drop corresponding to overlimiting currents. In this case, the SCR thickness increases sharply in comparison with the thickness of the equilibrium double layer. At such distances, the role of viscous forces decreases. Therefore, the main contribution to the development of overlimiting transport belongs to the electroosmosis of the second kind. This mode is similar to the Dukhin-Mishchuk mode described above. Nevertheless, it differs in that in the presence of the forced flow, the tangential force necessary for the occurrence of electroconvection arises due to the inhomogeneity of the longitudinal distribution of concentration, and not due to the electrical inhomogeneity of the surface.

For systems with forced flow at the threshold potential drop, *V*_cr1_ (Figure 1), single electroconvective vortices rotating in the same direction are formed in the region near the membrane surface (Figure 3) [17,19]. Vortices mix the electrolyte solution in the area near the membrane, which partially destroys the depletion layer and provides the regime of overlimiting current. Due to the forced flow, the vortices move along the solution/membrane interface towards the channel outlet. This movement of the vortices causes current density fluctuations on the CVC [19]. As the potential drop increases, the size of the vortices increases; at a certain potential drop (*V*_cr2_ on Figure 1) single vortices transform into large vortex complexes consisting of several vortices rotating in opposite directions [19,33]. As a result, the amplitude of the current density oscillations (or potential drop) increases and oscillations become chaotic [19]. P. Magnico investigated the role of electroconvective vortices in the fluid motion using the Lagrangian approach [33]. In this way, trajectories were constructed that reflect the ejection from the mixing layer, trapping by a growing vortex or merging vortices.

The electrical regime in membrane devices as a rule is determined in two ways: potentiodynamic (PD), when a potential drop in the system is set (constant, linearly increasing, periodically changing in time, etc.), and galvanodynamic (GD) when the current density is set (constant, linearly increasing, periodically changing in time, etc.).

Theoretical studies of transport processes taking into account the formation of the extended SCR and the development of electroconvection in membrane systems were mainly carried out for the PD regime using the equations of Navier-Stokes, Nernst-Planck and Poisson for the electric field potential [14,15,16,17,18,19,28,32,33]. The description of the GD regime caused difficulties associated with the absence of a differential equation for the current density. One approach to describing the ion transport in the membrane system in the GD regime is the decomposition of the system of Nernst-Planck and Poisson equations based on the assumption of local electroneutrality of the electrolyte solution [43,44]. In this approach, the distribution of a current density in the system is obtained using the electric current stream function. However, approaches based on the local electroneutrality assumption do not allow taking explicitly into account the effect of the SCR, which is formed at the solution/membrane boundary. Recently, these difficulties have been overcome using an approach involving the solution of the Poisson equation with a boundary condition determining the potential gradient through the current density [45,46].

This article presents numerical calculations of the CVCs and the hydrodynamic response of the electrolyte solution in flow-through membrane systems in the PD and GD regimes of the electric field. The structure of the electroconvective vortex layer is compared for these modes. For the first time, the hysteresis amplitude is calculated for flow-through systems in the PD and GD regimes.

## 2. Mathematical Models

CVCs were calculated:

(1) for the PD regime, when the potential drop, Δφ, is set to increase from 0 to a certain value, then to decrease from this value to 0:

(1) $$\Delta \varphi =\left\{ \begin{array}{ll} \alpha t, & t\leq t_{1},\\ 2\alpha t_{1}-\alpha t, & t>t_{1}, \end{array}\right.$$

where *α* > 0 is the potential sweep speed, *t*_1_ is the point in time at which the regime of the increasing potential drop is replaced by decreasing regime.

(2) for the GD regime, when the average current density, $i_{av}$, is set to increases from 0 to a certain value, then to decrease from this value to 0:

(2) $$i_{av}=\left\{ \begin{array}{ll} \beta t, & t\leq t_{2},\\ 2\beta t_{2}-\beta t, & t>t_{2}, \end{array}\right.$$

where *β* > 0 is the sweep speed of the current density, *t*_2_ is the point in time at which the regime of increasing current density is replaced by decreasing.

**Figure 4:**
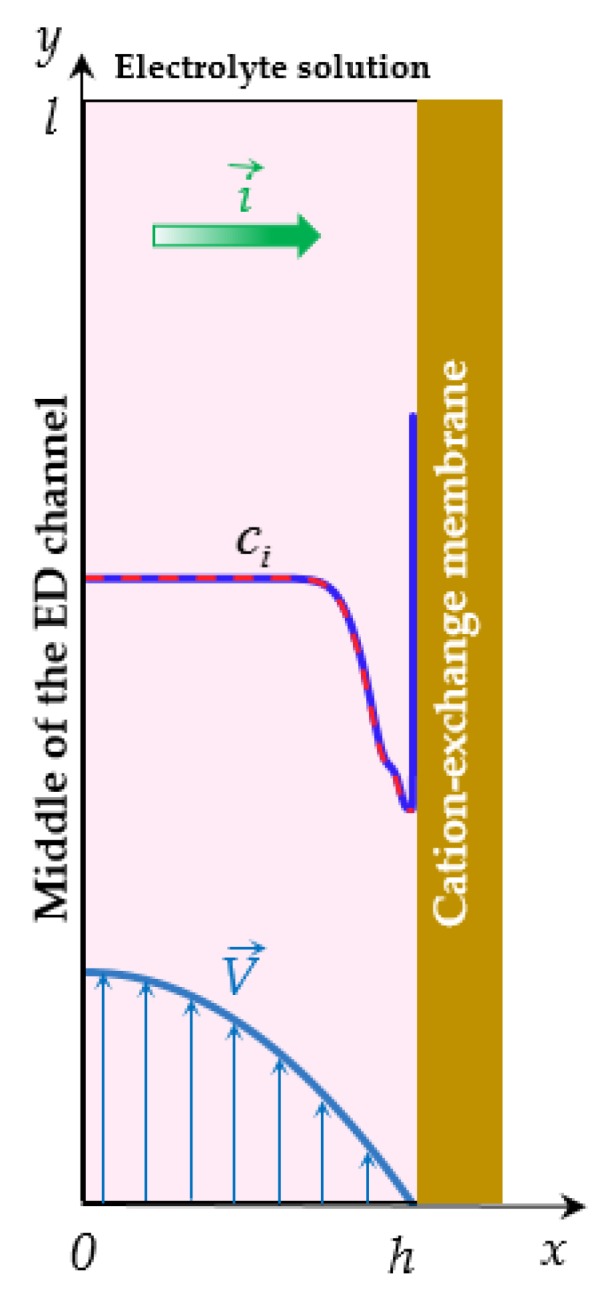
Scheme of the system under consideration: half of the desalination electrodialysis (ED) cell adjacent to CEM. Schematic concentration profiles of cations (*c*_1_, solid line) and anions (*c*_2_, dashed line), direction of the electric current $\vec{i}$, forced flow velocity $\vec{V}$ are shown.

The calculations are based on the 2D mathematical models of the overlimiting transfer enhanced by electroconvection in a flow-through ED cell for the PD [19,47] and GD [46] regimes. To simplify the numerical solution, we consider the processes in half of the ED channel at the surface of the cation-exchange membrane (CEM), Figure 4. Let *x* and *y* be the transverse and longitudinal coordinates, respectively; *x* = 0 relates to the middle of the ED channel, *x* = *h* is the electrolyte solution/CEM interface; *y* = 0 corresponds to the inlet and *y* = *l* to the outlet of the channel.

### 2.1. Formulation for the PD Regime

The non-stationary process of transfer of binary electrolyte ions in membrane systems in the absence of chemical reactions, with taking into account electroconvection, is written as follows [14,15,16,17,18,19]:

(3) ∂V→∂t+(V→∇)V→=−∇p+1ReΔV→+KelΔφ∇φ, divV→=0

(4) $${\vec{j}}_{i}=-z_{i}D_{i}c_{i}\nabla \varphi -D_{i}\nabla c_{i}+Pec_{i}\vec{V},\;i=1,\;2$$

(5) $$\frac{\partial c_{i}}{\partial t}=-\frac{1}{Pe}\;\mathrm{div}\;{\vec{j}}_{i},\;i=1,\;2$$

(6) $$-\varepsilon \Delta \varphi =z_{1}c_{1}+z_{2}c_{2},$$

(7) $$\vec{i}=z_{1}{\vec{j}}_{1}+z_{2}{\vec{j}}_{2}-\varepsilon \;Pe\;\frac{\partial }{\partial t}(\nabla \phi )$$

Equations (3)–(7) are given in dimensionless form. We scale time, *t*, by the value $h/V_{0}$; spatial coordinates, *x* and *y*, by the thickness of the considered region *h* (half of the ED channel thickness); velocity, $\vec{V}$, by the average velocity of the forced flow $V_{0}$; pressure, *p*, by the value $\rho V_{0}^{2}$; concentration of the i-th ion, *c_i_*, by the electrolyte concentration in the bulk solution *c*_0_; electric potential,φ, by the value RT/F; individual ion diffusion coefficients, *D*_1_ and *D*_2_, by the electrolyte diffusion coefficient $D=D_{1}D_{2}\left(z_{1}-z_{2}\right)/\left(D_{1}z_{1}-D_{2}z_{2}\right)$; current density, $\vec{i}$, by the value $Dc_{0}F/h$; ion flux ${\vec{j}}_{i}$ by the value $Dc_{0}/h$. Here $Re=V_{0}h/\nu$ is the Reynolds number, $Pe=V_{0}h/D$ is the Peclet number, $\varepsilon =RT\varepsilon_{0}\varepsilon_{r}/\left(c_{0}F^{2}h^{2}\right)=2{\left(L_{D}/h\right)}^{2}$ and $K_{el}=\varepsilon_{0}\varepsilon_{r}R^{2}T^{2}/\left(\rho_{0}V_{0}^{2}F^{2}h^{2}\right)$ are the dimensionless parameters; $z_{i}$ is the charge number of the i-th ion; *F* is the Faraday constant; *R* is the gas constant; *T* is the absolute temperature; $\varepsilon_{0}$ is the dielectric permittivity of vacuum; $\varepsilon_{r}$ is the solution relative permittivity (assumed constant); $\rho_{0}$ is the solution density (assumed constant), ν is the kinematic viscosity.

$\vec{V}$, *p*, ${\vec{j}}_{1}$, ${\vec{j}}_{2}$, *c*_1_, *c*_2_, φ, *i*_x_, *i*_y_ are unknown function of *t*, *x* and *y*. The Navier-Stokes equations, Equations (3), describe the velocity field under the action of the forced flow and the electric body force. The equations of Nernst-Planck, Equations (4), material balance, Equations (5), and Poisson, Equation (6), describe the ion concentration and potential fields. Equation (7) is a formula for the total current density, including the conduction current, ${\vec{i}}_{c}=z_{1}{\vec{j}}_{1}+z_{2}{\vec{j}}_{2}$, and displacement current, ${\vec{i}}_{d}=-\varepsilon \;Pe\;\frac{\partial }{\partial t}(\nabla \varphi )$. For the calculations of this article, the displacement current, *i_d_,* is negligible (less than 10^−7^).

The system of Equations (3)–(7) is supplemented by the boundary conditions [19,47]. At the channel inlet (*x* ∈ [0, *h*], *y* = 0), the velocity profile is parabolic and satisfies Poiseuille’s law (expressions for half of the ED channel):

(8) $$V_{x}\left(x,0,t\right)=0,\;V_{y}\left(x,0,t\right)=1.5(1-x^{2}).$$

In model from [19], the condition of uniform distribution along *x* for ion concentration at the channel inlet is accepted:

(9) $$c_{i}\left(x,0,t\right)=1,\;i=1,\;2.$$

In this paper, instead of condition (9), the Danckwerts’ boundary condition is used, which determines that arrival rate of ions into the channel is equal to the rate with which they cross the plane *y* = 0 by the combination of flow, electromigration, and diffusion [48]:

(10) $$\left(-z_{i}D_{i}c_{i}\nabla \varphi -D_{i}\nabla c_{i}+Pe\;c_{i}\vec{V}\right)\left(x,0,t\right)=Pe\;c\prime \;\vec{V},i=1,\;2,$$

where c′=1 is the input electrolyte concentration. The advantage of this condition in comparison with condition (9) is the absence of a special feature of the distribution of ion concentration near the point (*h*, 0). At the numerical implementation with condition (10), the accuracy of fulfilling the condition that the tangential current density through the inlet vanishes, *i_y_*(*x*,0,*t*) = 0, is higher than with condition (9) (Appendix A).

The condition for the electric potential is obtained from Equations (4) and (7) considering the zero tangential current density through the inlet, *i_y_*(*x*,0,*t*) = 0, (the tangential component of the displacement current, $i_{d\;y}$, is negligible):

(11) $$\frac{\partial \varphi }{\partial y}\left(x,0,t\right)=-\frac{1}{z_{1}^{2}D_{1}+z_{2}^{2}D_{2}}\left(z_{1}D_{1}\frac{\partial c_{1}}{\partial y}+z_{2}D_{2}\frac{\partial c_{2}}{\partial y}\right)$$

At the channel outlet (*x* ∈ [0, *h*], *y* = *l*) the velocity profile is again parabolic; the sum of diffusion and migration tangential components of the cation (*i* = 1) and anion (*i* = 2) fluxes is zero; the tangential derivative of the potential is set to be zero:

(12) $$V_{x}\left(x,l,t\right)=0,\;V_{y}\left(x,l,t\right)=1.5(1-x^{2})$$

(13) $$\left(-\frac{\partial c_{i}}{\partial y}-z_{i}c_{i}\frac{\partial \varphi }{\partial y}\right)\left(x,l,t\right)=0,\;i=1,2$$

(14) $$\frac{\partial \varphi }{\partial y}\left(x,l,t\right)=0.$$

At *x* = 0, *y* ∈ [0, *l*] (middle of the ED channel) the following conditions are applied:

(15) $$V_{x}\left(0,y,t\right)=0,\;V_{y}\left(0,y,t\right)=1.5$$

(16) $$c_{i}\left(0,y,t\right)=1,\;i=1,\;2$$

(17) φ(0,y,t)=0.

At *x* = 1, *y* ∈ [0, *l*] (the solution/membrane interface), the no-slip condition (18) is applied; the counterion concentration, *c*_1_, is set as a constant value *N_c_* greater than the bulk solution concentration, Equation (19), [25]; continuous flow of co-ions, Equation (20); the potential drop is set, Equation (21):

(18) $$V_{x}\left(1,y,t\right)=0,\;V_{y}\left(1,y,t\right)=0$$

(19) $$c_{1}\left(1,y,t\right)=N_{c}$$

(20) $$\left(-D_{2}\frac{\partial c_{2}}{\partial x}-z_{2}D_{2}c_{2}\frac{\partial \varphi }{\partial x}\right)\left(1,y,t\right)=\frac{\left(1-T_{1}\right)}{z_{2}}i_{x}\left(1,y,t\right)$$

(21) φ(1,y,t)=Δφ

The potential drop, Δφ, is given by Equation (1).

Thus, the formulation of the model for the PD regime includes the system of Equations (3)–(7) and boundary conditions (8), (10)–(21). The average over the channel length current density is calculated as [46]:

(22) $$i_{av}=\frac{1}{l}\int_{0}^{l}\int_{0}^{1}i_{x}dxdy$$

### 2.2. Formulation for the GD Regime

To describe the GD regime, Equations (3)–(6) and boundary conditions (8), (10)–(20) are used similarly to PD regime, but there are two differences. First, at the boundary *x* = 1, *y* ∈ [0, *l*] (solution/membrane interface), instead of condition (21), normal to the membrane surface component of the electric field strength is specified as function of the electric current density [46]:

(23) $$\frac{\partial \varphi }{\partial x}\left(1,y,t\right)=-\left(\frac{\left(i_{x}+\varepsilon \;Pe\frac{\partial^{2}\varphi }{\partial x\partial t}+z_{1}D_{1}\frac{\partial c_{1}}{\partial x}+z_{2}D_{2}\frac{\partial c_{2}}{\partial x}\right)}{z_{1}^{2}D_{1}c_{1}+z_{2}^{2}D_{2}c_{2}}\right)(1,y,t)$$

Condition (23) was obtained from Equations (4) and (7) [46,49].

Secondly, an additional equation is introduced to determine the distribution of current density, which is required by the boundary condition (23). For this purpose, the method of electric current flow function is used [43,44,45,46]. According to this method, the electric current stream function, η, is determined:

(24) $$i_{x}=\frac{\partial \eta }{\partial y},\;i_{y}=-\frac{\partial \eta }{\partial x}$$

Then the equation and boundary conditions for η are introduced to the mathematical formulation of the model [45,46]:

(25) $$\begin{array}{l} \Delta \eta =-\left(\left(z_{1}^{2}D_{1}\frac{\partial c_{1}}{\partial y}+z_{2}^{2}D_{2}\frac{\partial c_{2}}{\partial y}\right)\frac{\partial \varphi }{\partial x}-\left(z_{1}^{2}D_{1}\frac{\partial c_{1}}{\partial x}+z_{2}^{2}D_{2}\frac{\partial c_{2}}{\partial x}\right)\frac{\partial \varphi }{\partial y}\right)+\\ +Pe\left(z_{1}\frac{\partial c_{1}}{\partial y}+z_{2}\frac{\partial c_{2}}{\partial y}\right)V_{x}-Pe\left(z_{1}\frac{\partial c_{1}}{\partial x}+z_{2}\frac{\partial c_{2}}{\partial x}\right)V_{y}+Pe\left(z_{1}c_{1}+z_{2}c_{2}\right)\left(\frac{\partial V_{x}}{\partial y}-\frac{\partial V_{y}}{\partial x}\right), \end{array}$$

(26) $$\frac{\partial \eta }{\partial x}\left(0,y,t\right)=0,\;\frac{\partial \eta }{\partial x}\left(1,y,t\right)=0,\;\eta \left(x,0,t\right)=0,\;\eta \left(x,l,t\right)=i_{av}l$$

**Figure A2:**
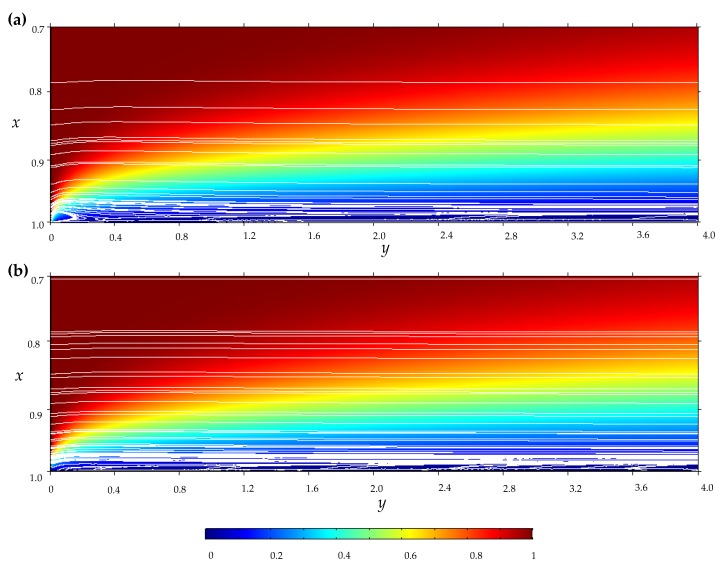
Distribution of cation concentration (the magnitude is shown by different colors), solution streamlines (white lines) in the area at the membrane surface. Calculation for the PD regime at ∆φ = 19.5 with condition (9) (**a**) and with Danckwerts’ condition (10) (**b**). To improve the visibility of the electroconvective vortex layer, the scale along the *x* axis is set larger than the *y* axis, thus the shape of the vortices is deformed.

The boundary conditions (26) were derived under the simplifying assumption that the current through the channel outlet *i_y_*(*x*,*l*,*t*) ≈ 0 (due to its smallness, Figure A2). Therefore, average current density, *i*_av_, can be used as a parameter determining the electrical regime in the system:

(27) $$i_{av}=\frac{1}{l}\int_{0}^{l}i_{x}(0,y,t)dy=\frac{1}{l}\int_{0}^{l}i_{x}(1,y,t)dy$$

Thus, current density *i_x_* in boundary condition (23) is determined by Formula (24).

Thus, the formulation of the model for the GD regime includes the system of Equations (3)–(6), (25) and boundary conditions (8), (10)–(20), (23) and (26).

### 2.3. Numerucal Implementation

Numerical solutions were found by the finite element method using Comsol Multiphysics 5.1 software package. The results presented below were obtained using a non-uniform unstructured triangular computational grid consisting of about 55,000 elements. The density of the mesh elements was increased near the solution/membrane boundary: 1000 elements were set using the “Distribution” node. The influence of the quality of the computational mesh was tested by comparing solutions for two meshes consisting of about 41,000 elements (when “Distribution” node set 700 elements on the solution/membrane boundary) and 55,000 elements (with 1000 elements on the boundary). The difference in the values of the threshold potential drop of the transition to the overlimiting current mode (both in increasing and decreasing regimes) did not exceed 2%.

The following modules are used to implement the model for the GD regime: “Laminar flow” for the Navier-Stokes Equation (3); “Transport of Diluted Species” for the anions and cations concentrations fields, Equations (4) and (5); “Poisson’s equation” for the electric potential fields, Equation (6); “General form PDE” for the electric current stream function, Equation (25). For spatial discretization of the concentration, potential, and the electric current stream function fields, the quadratic Lagrange interpolation functions are used. The “Laminar flow” module has the “P2 + P1” discretization that means second order elements for the velocity components and linear elements for the pressure field [50].

For time-depended calculations a segregated node with implicit time-stepping method BDF (backward differentiation formulas) is used [50]. One segregated iteration consists of executing two segregated step: in the first step, concentration, potential and electric current stream function are calculated; on the second, speed and pressure are calculated. At each step, the multifrontal massively parallel sparse direct solver (MUMPS) method [50] is used.

The time step is automatically determined by the solver so that the requirement for the relative tolerance is met (its value was set equal to 10^−8^). With a decrease in the relative tolerance by a factor of 10, the change in the threshold potential drop of the transition to the overlimiting mode did not exceed 1%.

The implementation of the PD regime is similar to the described for the GD regime with the difference that the equation for the electric current stream function (25) is excluded from the calculation process and the boundary condition for the potential (23) changes to (21).

## 3. Results

### 3.1. Parameters Used in Computations

The results of simulation presented here are obtained for a flow-through ED cell (Figure 4) in the case of dilute NaCl solutions. The dimensionless parameters *ε* = 3.05 × 10^−8^, *Pe* = 589, *Re* = 1.07, *K*_el_ = 5.23 × 10^−4^, which correspond to the following system parameters: the thickness of the considered region *h* = 0.5*H*, where *H* = 0.5 × 10^−3^ m is the intermembrane distance; the channel length *l* = 10^−3^ m; the average velocity of forced flow *V_0_* = 3.8 × 10^−3^ m/s; the electrolyte solution density *ρ_0_* = 1002 kg/m^3^; the kinematic viscosity ν = 0.89 × 10^−6^ m^2^/s; the input concentration of the electrolyte solution of NaCl *c*_0_ = 0.1 mol/m^3^; the temperature *T* = 298 K; the diffusion coefficients of cations *D*_1_ = 1.33 × 10^−9^ m^2^/s and anions *D*_2_ = 2.05 × 10^−9^ m^2^/s; the cation transport number in the membrane *T*_1_ = 0.972 and that in the solution *t*_1_ = 0.395; the ion charge numbers *z*_1_ = 1, *z*_2_ = −1. To simplify the numerical solution, the ratio of the counterion concentration at the solution/CEM boundary to its value in the bulk solution *N*_c_ was taken as *N*_c_ = 1. This value is less than in real systems, however, as Urtenov et al. [51] have shown, when *N*_c_ ≥ 1, the value *N*_c_ does not essentially affect the distribution of concentrations and potential in the extended SCR.

The sweep speeds of the potential drop (*α* = 0.0064) and average current density (*β* = 0.0003) are chosen sufficiently small and the solution can be considered quasi-stationary, that is, their values do not affect the CVCs trend.

### 3.2. Current-Voltage Curves

**Figure 5:**
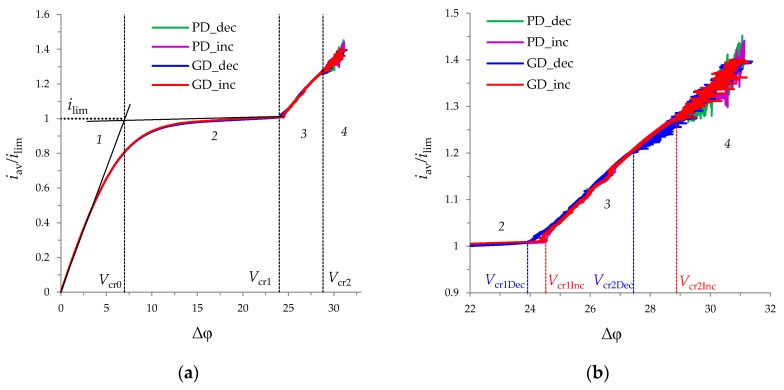
(**a**) CVCs calculated for the potentiodynamic (PD) (increasing Δφ—purple line, decreasing Δφ—green line) and galvanodynamic (GD) (increasing *i*_av_—red line, decreasing *i*_av_—blue line) regimes. The dotted line shows the limiting current density, *i*_lim_, calculated using Leveque’s Equation (28). The dashed lines *V*_cr0_, *V*_cr1_, *V*_cr2_ indicate changes in the CVC regions: underlimiting current **1**, plateau of the limiting current **2**, overlimiting **3**, overlimiting with chaotic oscillations **4**. (**b**) enlarged fragment of (**a**).

Figure 5 shows the CVCs calculated for the GD and PD regimes. All CVCs have a linear initial part (denoted by 1 in Figure 5a), a sloping plateau (2 in Figure 5a), and an overlimiting current (3,4 in Figure 5a), which qualitatively corresponds to the existing experimental [5,7,9,13] and theoretical [16,19,31] studies about the CVCs of membrane systems. Note that the limiting current density of the calculated CVCs, determined by the point of intersection of the tangents drawn to the initial part and to the sloping plateau of the curve is close to *i*_lim_, calculated using Leveque’s Equation (28) (values differ by less than 2%) [47]:

(28) $$i_{\lim}=\frac{1}{T_{1}-t_{1}}\left(1.47{\left(\frac{4h^{2}V_{0}}{lD}\right)}^{1/3}-0.2\right)$$

**Figure 6:**
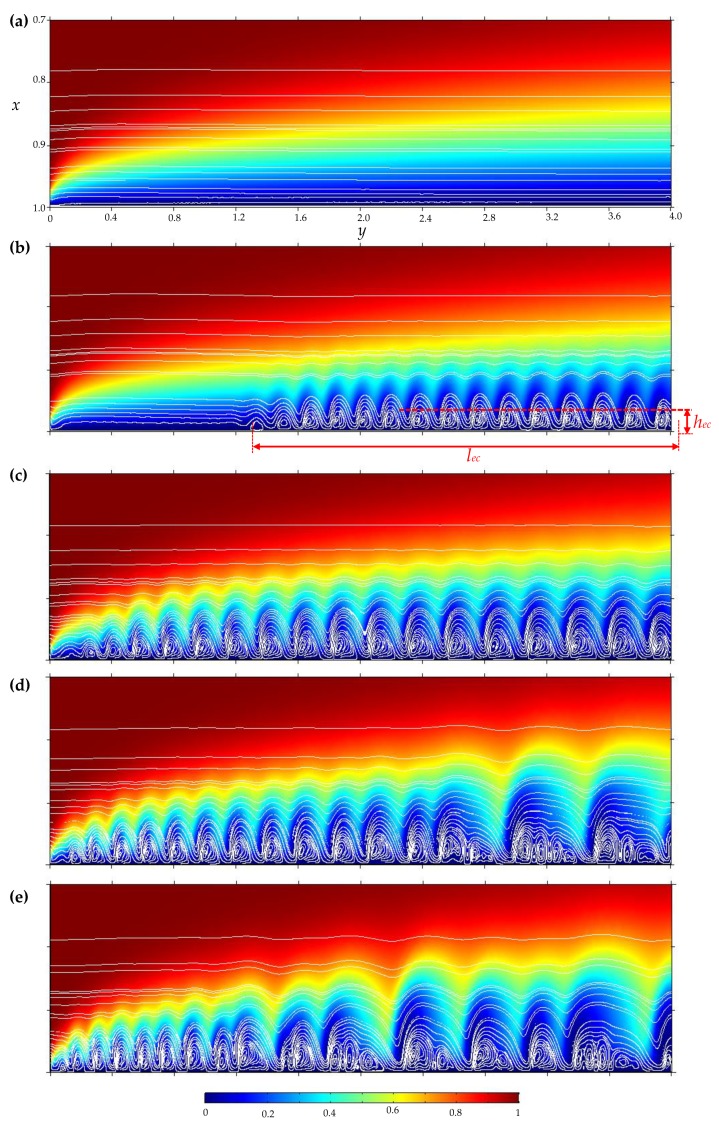
Distribution of cation concentration (the magnitude is shown by different colors), solution streamlines (white lines) in the area at the membrane surface. Calculation for the increasing GD regime at *i*_av_/*i*_lim_ = 1 (**a**), 1.1 (**b**), 1.25 (**c**), 1.3 (**d**), 1.35 (**e**). To improve the visibility of the electroconvective vortex layer, the scale along the *x* axis is set larger than the *y* axis, thus the shape of the vortices is deformed.

At the underlimiting and limiting current modes (regions *1* and *2* on Figure 5a, respectively) of the CVCs calculated for PD and GD regimes coincide with high accuracy (the difference is less than 0.01*i*_lim_). In these modes (at current densities *i*_av_/*i*_lim_ ≤ 1 or potential drop Δφ < *V*_cr1_), electroconvective vortices are not observed in the fluid flow (Figure 6a).

At the overlimiting current modes of the CVCs calculated for the both regimes single electroconvective vortices rotating in the same direction (for region *3* on Figure 5a; Figure 6b,c) and large vortex complexes consisting of several vortices rotating in opposite directions (for region *4* on Figure 5a; Figure 6d, e) are formed in the region near the membrane surface. Movement of the vortices causes current density fluctuations in the PD regime and potential drop fluctuations in the GD regime (regions *3* and *4* on Figure 5a). At the same time, the trends of the overlimiting current regions of the CVCs in both regimes approximately coincide (Figure 5b).

### 3.3. Electroconvective Vortex Layer

To quantitatively describe the electroconvection vortex layer, parameters such as the thickness, *h_ec_*, and length, *l_ec_*, and density of vortices, *d_ec_*, of this layer were determined. For systems with forced flow, the vortex sizes are not stable and depend on its position in the channel [17,19,47]; therefore, at a given point in time the thickness, *h_ec_*, was determined as the distance from the membrane surface to the farthest edge of the closed streamline forming the biggest vortex [47] (Figure 6b). At each moment of time, the electroconvective vortex layer is a set of successive vortices and vortex structures. Wherein, this layer appears in the region at the channel outlet. Therefore, length, *l_ec_*, was defined as the distance from the outlet to the farthest edge of the closed streamline forming the first (from the inlet) vortex (Figure 6b). Thus, *h_ec_* and *l_ec_* characterize the dimensions of the electroconvective vortex layer, that is the maximum transverse dimension of the biggest vortex and the length of the entire layer at a moment in time. Another important parameter characterizing the electroconvective vortex layer is the density of vortices, *d_ec_*; that is, the number of vortices per unit of length.

**Figure 7:**
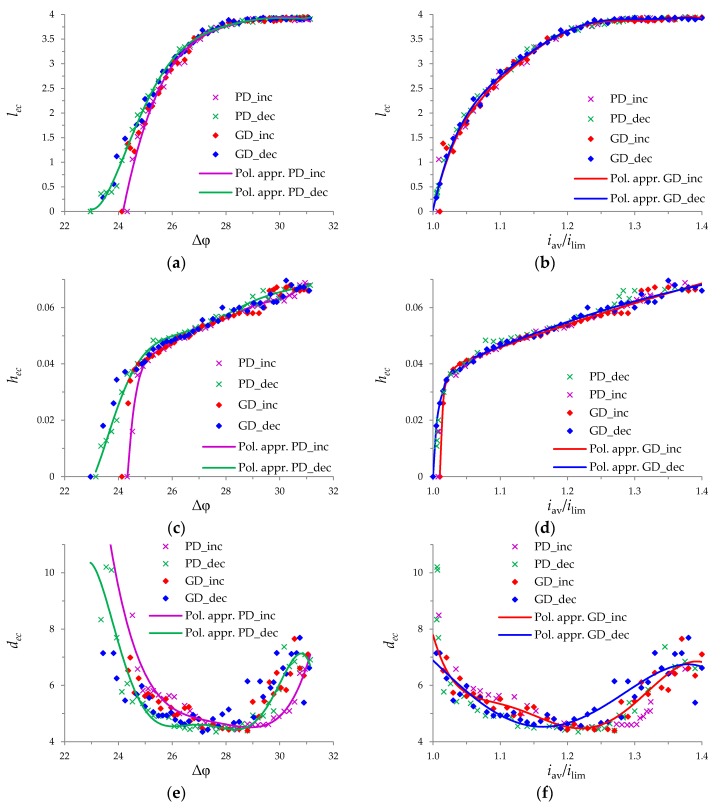
Dependences of the length, *l_ec_*, thickness, *h_ec_*, and vortex density, *d_ec_*, of the electroconvective vortex layer on the potential drop, respectively (**a**), (**c**), (**e**), and current density (**b**), (**d**), (**f**). Calculations for the PD regime are indicated by crosses and for the GD regime by rhombuses. The solid lines (Pol. appr.) indicate polynomial approximation of the corresponding data.

The values of *h_ec_*, *l_ec_*, *d_ec_*, were calculated for the PD regime for the potential drop Δφ = 23.4, 23.6, …, 31 (the results are indicated by crosses and trend lines in Figure 7a,c,e). Figure 7b,d,f show values of *h_ec_*, *l_ec_*, *d_ec_*, calculated for the GD regime at the current density *i*_av_/*i*_lim_ = 1, 1.01, …, 1.4. The increase in the length of the electroconvective vortex layer, *l_ec_*, is limited by the moment (Δφ ≈ 28 or *i*_av_/*i*_lim_ ≈ 1.24), when this layer occupies almost the entire length of the channel (*l* = 4), Figure 7a,b. The thickness of the electroconvective vortex layer, *h_ec_*, increases approximately linearly with increasing potential drop (or current density) everywhere in the considered range of Δφ (or *i*_av_) values, except for the initial region of rapid growth at Δφ ≈ *V*_cr1_ (or *i*_av_/*i*_lim_ ≈ 1), Figure 7c,d. Saturation of the thickness, *h_ec_*, is not observed.

Figure 7e,f show a decrease in the vortices density, *d*_ec_, in the range of the potential drop (or current density) corresponding to a rapid increase in the size of the electroconvective vortex layer; and the increase density, *d*_ec_, in the range of the development of the large vortex complexes consisting of several vortices rotating in opposite directions (Figure 6d,e).

The described behavior of the system is characteristic of both the PD and GD regimes, both for increasing and decreasing cases. To compare the parameters of the electroconvective vortex layer in the GD and PD regimes, the average current densities, $\bar{i_{av}}$ corresponding to Δφ= 23.4, 23.6, …, 31 for the PD regime and the average values of the potential drop, $\bar{\Delta \varphi }$, corresponding to *i*_av_/*i*_lim_ = 1, 1.01, … 1.4 were calculated for GD regime. Figure 7 also show the dependences *h_ec_*, *l_ec_*, *d_ec_*, on $\bar{\Delta \varphi }$, calculated for the GD regime and on $\bar{i_{av}}$, calculated for the PD regime. Figure 7 shows that the dependences of the parameters of the electroconvective vortex layer on the potential drop and current density in the PD and GD regimes are approximately the same.

### 3.4. Comparison of Increasing and Decreasing Regimes (Hysteretic Behavior)

The differences in the CVCs calculated with increasing and decreasing potential drop (or average current density) are manifested in the overlimiting current mode both in the PD and GD cases.

The critical potential drop of the transition to the overlimiting current mode in the increasing regime, *V*_cr1Inc_, is larger than the corresponding value in the decreasing regime, *V*_cr1Dec_ (Figure 5b): V_cr1Inc_ ≈ 24.52 and V_cr1Dec_ ≈ 23.92 (these values approximately coincide for the PD and DG regimes). The hysteresis amplitude (∆*V*_cr1_ = *V*_cr1Inc_ − V_cr1Dec_ ≈ 0.6) is less than the difference in the potential drops correspond for the appearance and disappearance of vortices (determined by the values of *h_ec_*, *l_ec_*), which is about 1.2. This is due to the fact that the transition between the limiting and overlimiting modes on CVC appears only when the thickness *h_ec_* exceeds approximately 0.02. The electroconvective vortex layer of the smaller thickness only causes fluctuations of small amplitude in the CVC.

The calculations in this article confirm the existence of the hysteretic behavior for flow-through channels in both PD and GD regimes. In the transition region between the limiting and overlimiting modes at the fixed potential drop in the decreasing regime, the length, *l_ec_*, and thickness, *h_ec_*, of the electroconvective vortex layer are greater than in the increasing regime (Figure 7a,c). At the fixed average current density, the length, *l_ec_*, and thickness, *h_ec_*, of the electroconvective vortex layer in the decreasing and increasing regimes approximately coincide (Figure 7b,d). In this region (Δφ ≈ *V*_cr1_, *i*_av_/*i*_lim_ ≈ 1), the density of vortices, *d_ec_*, is higher in the increasing regime compared to the decreasing (Figure 7e,f).

In addition, the critical potential drop of the transition to chaotic oscillations in the increasing regime, *V*_cr2Inc_, is also larger than the corresponding value in the decreasing regime, *V*_cr2Dec_ (Figure 5b): *V*_cr2Inc_ ≈ 28.88 and *V*_cr2Dec_ ≈ 27.44 for GD case; *V*_cr2Inc_ ≈ 30.36 and *V*_cr2Dec_ ≈ 28.61 for PD case. In the region of chaotic oscillations of the CVCs, the length and thickness of the electroconvective vortex layer oscillate in the same range for the increasing and decreasing regimes, but the density of vortices in the decreasing regime is higher. This is due to the fact that vortex complexes consisting of many vortices are maintained at the lower potential drop in the decreasing regime compared to increasing one.

## 4. Conclusions

On the CVCs calculated for the PD and DG regimes, four main current modes can be distinguished: underlimiting, limiting, overlimiting, and chaotic overlimiting. The influence of the electric field regime is manifested in the overlimiting current modes when a significant electroconvection vortex layer develops in the channel. The slipping of vortices along the membrane surface under the action of the forced flow leads to fluctuations in the current density at the PD regime and oscillations in the potential drop at the GD regime. The trend lines of the overlimiting sections of the CVCs for the PD and GD regimes are approximately the same, since the values of the parameters of the electroconvective vortex layer at the same values of the potential drop (or current density) in these modes are quite close.

At the fixed potential drop, the length and thickness of the electroconvective vortex layer in the decreasing regime (PD or GD) is greater than in an increasing one. This leads to the formation of a hysteresis loop in the transition region between the limiting and overlimiting regions of the CVCs. There is also a difference in the critical potential drop of the transition to the chaotic oscillations mode in the increasing and decreasing regimes.

Thus, the development of electroconvection determines the influence of the electric field regime on the processes of ion transfer in membrane systems.

## Figures and Tables

**Figure A1:**
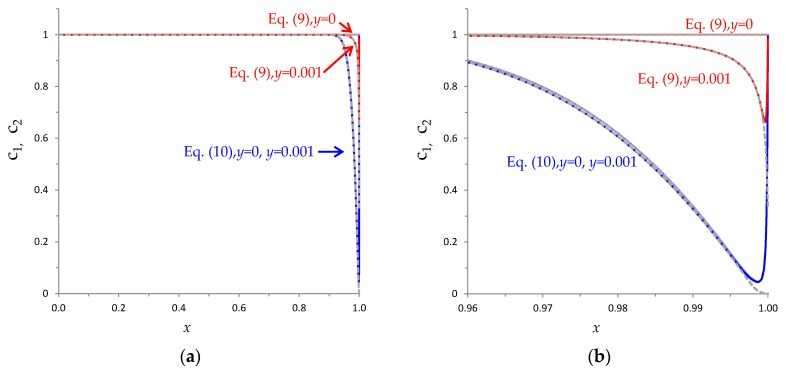
(**a**) Concentration profiles of cations (*c*_1_, solid lines) and anions (*c*_2_, dashed lines) in sections *y* = 0 and *y* = 0.001. Calculation for the PD regime at ∆φ = 19.5 with condition (9) (red lines) and with Danckwerts’ condition (10) (blue lines). (**b**) enlarged fragment of (**a**).

**Figure A3:**
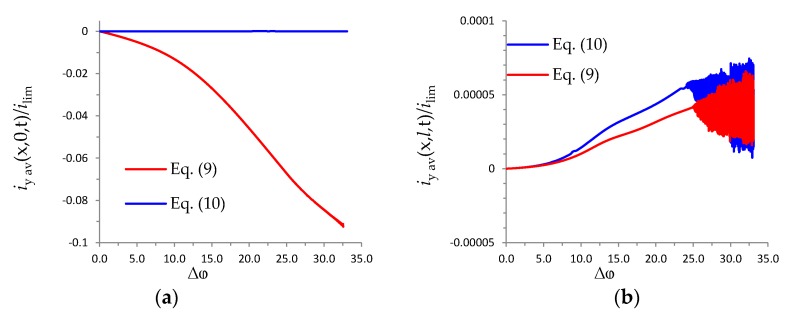

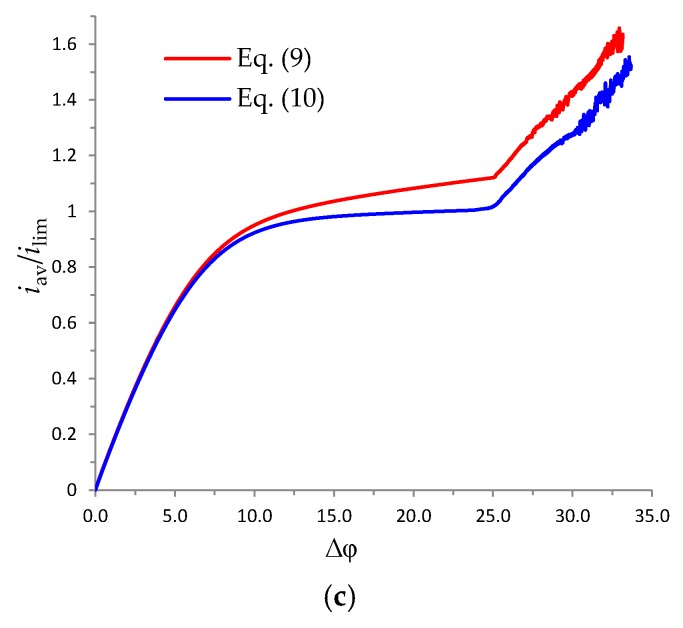
Average tangential current density through the inlet (**a**) and outlet (**b**); CVCs (**c**). The calculation results with condition (9) (red lines) and Danckwerts’ condition (10) (blue lines) for the PD regime at *α* = 0.025, other parameters as in p. 3.1.

## Appendix A

Figure A1 shows the concentration profiles for the channel inlet, *y* = 0, and at a short distance from it, *y* = 0.001, calculated with the condition of the uniform ion distribution (9) and with Danckwerts’ condition (10) at ∆φ = 19.5. In the first case, the concentration profiles vary significantly in the longitudinal direction: for the section *y* = 0.001, they are lower than for *y* = 0; in the second case, concentration profiles practically coincide (maximal difference less than 0.01). As a result, in the calculation with condition (9), a stationary vortex is formed at the inlet at ∆φ < *V*_cr1_, that is, earlier than that for the rest of the channel (Figure A2). When condition (10) is used, vortices at the inlet appear at ∆φ > *V*_cr1_ during the growth of the electroconvective vortex layer, the formation of which begins at the outlet (Figure 6e).

In the Figure A3a,b, the dependences of the average tangential current density through the inlet, *i*_y av_(*x*,0*,**t*), and outlet, *i*_y av_(*x*,*l,**t*), boundaries on time are shown. In the considered range of the potential drop, the current *i*_y av_(*x*,0*,**t*), calculated with condition (10), does not exceed 5 × 10^−5^*i*_lim_; for condition (9), this value reaches 0.09 *i*_lim_. As a result, at the calculations with condition (10), the plateau angle of the limiting current decreases; the overlimiting region of the CVC lies lower (Figure A3a).
